# Gastroesophageal Reflux, Sleep‐Disordered Breathing, and Outcomes in Patients With Idiopathic Pulmonary Fibrosis

**DOI:** 10.1155/carj/4228567

**Published:** 2025-12-28

**Authors:** Braden Ellis, Daniel Morris, Andrea Peterson, Isabella Marquetti, Luke Manietta, Mikal Borg, Stephen Halliday, Amy Malik, Nathan Sandbo, Ronald Gangnon, Christopher J. Francois, Mihaela Teodorescu

**Affiliations:** ^1^ Department of Medicine, University of Wisconsin School of Medicine and Public Health, Madison, Wisconsin, USA, uwm.edu; ^2^ Medical Service, William S. Middleton Memorial Veterans’ Affairs Medical Center, Madison, Wisconsin, USA; ^3^ Department of Population Health Sciences, University of Wisconsin School of Medicine and Public Health, Madison, Wisconsin, USA, uwm.edu; ^4^ Department of Biostatistics and Medical Informatics, University of Wisconsin School of Medicine and Public Health, Madison, Wisconsin, USA, uwm.edu; ^5^ Department of Radiology, Mayo Clinic, Rochester, Minnesota, USA, mayo.edu

**Keywords:** gastroesophageal reflux, idiopathic, obstructive, patient-centered outcomes, periodic limb movement, pulmonary fibrosis, sleep apnea, sleep-disordered breathing

## Abstract

**Background:**

Gastroesophageal reflux disease (GERD) and obstructive sleep apnea (OSA) may negatively impact idiopathic pulmonary fibrosis (IPF), but data on their concurrent contributions are lacking. We aimed to test the contributions of GERD and sleep‐disordered breathing (SDB) to IPF outcomes.

**Methods:**

We performed a cross‐sectional, exploratory study on subjects with IPF. Clinically established GERD diagnosis, questionnaires (Nocturnal GERD Symptom Severity and Impact Questionnaire [N‐GSSIQ], the NIH Patient‐Reported Outcomes Measurement Information System [PROMIS] sleep impairment and fatigue scales, and Short Form‐36 [SF‐36]), full pulmonary function tests (PFT), six‐minute walk test (6MWT), and nocturnal polysomnography (PSG) were obtained.

**Results:**

Among *n* = 24 subjects, 17 (71%) had clinically diagnosed GERD. N‐GSSIQ scores indicated a nocturnal burden, which was adversely related to sleep impairment (*p* = 0.010) and daytime fatigue (*p* = 0.001), tiredness (*p* = 0.026) and SF‐36 social functioning (*p* = 0.005), energy/fatigue (*p* = 0.015), pain (*p* = 0.030), and health change in the prior year (*p* = 0.035). From PSG, GERD correlated with worse sleep architecture (GERD diagnosis, all *p* < 0.05) and periodic leg movements index (PLMI) (N‐GSSIQ, *p* = 0.02). GERD was not associated with pulmonary or exercise physiology. Overall, apnea–hypopnea index (AHI) was (median [25% quartile, 75% quartile]) 18.2 (8.1, 27.8)/h, and 19 (79%) subjects had OSA (AHI ≥ 5/h), with most (15/19 [79%]) having moderate or severe disease. SDB measures adversely related to gas exchange and distance walked (all *p* < 0.05).

**Conclusions:**

A nocturnal burden of GERD was detected and related to sleep disruption, including PLMs, and to daytime complaints. SDB/OSA, of a severity known to have significant health consequences, was common; it was adversely related to pulmonary diffusion and exercise capacity. These findings call for comprehensive, early evaluation of GERD and OSA for improved IPF outcomes.

## 1. Introduction

Idiopathic pulmonary fibrosis (IPF) is a progressive disease characterized by proliferation of fibrotic tissue, leading to decrement in lung function and symptoms, including cough, dyspnea, fatigue, and limitations in daily activities [1]. Patients with IPF often present with comorbid gastroesophageal reflux disease (GERD), which features a bidirectional relationship with IPF [2]. The increased IPF‐related lung recoil coupled with an increase in negative intrathoracic pressures promotes lower and upper esophageal sphincter dysfunction, facilitating microaspirations of gastric fluid [2]. These, in turn, lead to repeated injury to the pulmonary alveolar epithelium, fibrotic remodeling of the parenchyma, and exacerbation of IPF [2]. Additionally, GERD may induce sleep disruption, which could affect daytime alertness and quality of life [3].

Obstructive sleep apnea (OSA) is highly prevalent among patients with IPF, where its treatment with positive airway pressure (PAP) may reduce IPF‐related mortality and improve patient‐centered outcomes [4]. Sleep‐disordered breathing (SDB), with its prototype OSA, is characterized by partial or complete cessation of breathing during sleep due to upper airway collapse, associated with increased work of breathing, desaturations, and sleep disruption that have significant health consequences [5].

Although both GERD and SDB can impact IPF outcomes and daytime symptoms, data concerning the concurrent impact of GERD and OSA on IPF progression are lacking, which is important because recognition of OSA lags behind that of GERD and other IPF comorbidities [4]. We aimed to assess the contribution of nocturnal GERD and related sleep disruption, and of SDB to patient‐centered and pulmonary outcomes in IPF. We hypothesized that nocturnal GERD and SDB will each correlate with adverse IPF outcomes. A preliminary report of this study was published in abstract form [6].

## 2. Study Design and Methods

This was an exploratory, ancillary, add‐on study to a broader aim, cross‐sectional, single‐visit study focused on the relationship of OSA with cardiopulmonary outcomes in IPF. The study was conducted at the James B. Skatrud Pulmonary and Sleep Research Laboratory, William S. Middleton Memorial Veterans’ Affairs Medical Center (VAMC) and was approved by the University of Wisconsin (UW)–Madison Health Sciences Institutional Review Board (HS‐IRB #2019‐0891, approval date December 19, 2019) and the VAMC Research and Development Committee (UW HS‐IRB #2021‐0959 and VA IRBNet #1638735; approval date March 17, 2022). All participants provided informed consent.

### 2.1. Study Population

Subjects aged 40–85 with a multidisciplinary‐established IPF diagnosis followed at UW Interstitial Lung Disease and VAH Pulmonary clinics were identified via electronic medical records (EMR) and/or daily clinic rosters. Inclusion criteria required being on antifibrotic medication (nintedanib or pirfenidone) at stable doses for at least 6 months prior to study participation, being free of exacerbation in the preceding 6‐week period, and for patients using PAP at home—which can impact the severity of OSA—being willing to hold therapy for 1 week prior to the sleep study. Subjects with an inability to perform required study procedures, any interfering medical, psychiatric, neuromuscular, or medications (i.e., narcotics, benzodiazepines, or barbitals), among other criteria, were excluded. Enrolled subjects were invited to the laboratory for one study visit.

### 2.2. Clinical Data Collected

Clinical data extracted from EMR included demographics (age and sex), an established GERD diagnosis and medications (such as proton‐pump inhibitors [PPIs], histamine H_2_‐receptor blockers [H_2_‐blockers], antacids, and surface agents), number of hospitalizations, unscheduled doctor visits and steroid bursts due to worsening respiratory status in the 12 months before enrollment, other comorbidities (including OSA), and, if applicable, PAP adherence data. These were verified with subjects at their visits, when physical examinations (including height, weight, and neck circumference) were performed.

### 2.3. Questionnaires

During the visit, subjects completed a battery of self‐administered questionnaires, which included (1) the Nocturnal Gastroesophageal Reflux Disease Symptom Severity and Impact Questionnaire (N‐GSSIQ), assessing nocturnal GERD symptoms, morning impact, and concern about nocturnal GERD [7]; (2) the National Institutes of Health Patient‐Reported Outcomes Measurement Information System (PROMIS) v1.0 Short Forms [8], including Sleep‐Related Impairment, Sleep Disturbance, Fatigue, Anxiety and Depression, and the RAND Short Form 36 Health Survey (SF‐36) Version 1.0 [8, 9]; and (3) the University of California at San Diego Shortness of Breath Questionnaire (SOBQ) [10]. Subjects also responded to individual dichotomous (Yes/No) questions on daytime sleepiness, fatigue, lack of energy, and tiredness and questions on lifetime healthcare utilization (number of hospitalizations, admissions to intensive care units [ICU], and need for mechanical ventilation) due to IPF.

The N‐GSSIQ is a self‐reported instrument assessing nocturnal GERD over the past 2 weeks. It was validated against GERD severity rated by both patients and their clinicians, in a large cohort [7]. The N‐GSSIQ contains 3 domains: Nocturnal GERD Symptom Severity (13 items), Morning Impact of Nocturnal GERD (2 items), and Concern about Nocturnal GERD (3 items). Each item of the Nocturnal GERD Symptom Severity and Morning Impact of Nocturnal GERD domains is scored on a six‐point Likert scale ranging from *none* to *very severe* and *none of the time* to *all of the time*, respectively. Items of concern about the Nocturnal GERD domain are scored on a five‐point Likert scale ranging from *not at all concerned* to *extremely concerned.* Each subscale score is calculated by the sum of each item score in the domain. The overall score is calculated as the mean of the subscale scores of the Nocturnal GERD Symptom Severity and Morning Impact of Nocturnal GERD domains only. Total score ranges from 0 to 37.5, with higher scores denoting worse control. No known validated threshold for control or minimal validated clinical difference has been published.

Subjects completed also the National Institutes of Health PROMIS v1.0 Short Forms [8], including Sleep‐Related Impairment 8a, Sleep Disturbance 8a, Fatigue 8a, Anxiety 8a, and Depression 8a. All NIH PROMIS v1.0 Short Forms have been validated within a large, general population sample as measures of core health‐related quality of life domains [11]. Each questionnaire contains eight items assessing symptoms with a 7‐day time window. Items are ranked on a five‐point Likert scale. For each questionnaire, a raw score is calculated as the sum of each item score and then is matched with the provided score conversion tables to compute a final t‐score for comparison purposes. Higher total t‐scores denote worse symptom burden.

The RAND SF‐36 Version 1.0 is a self‐reported, validated tool for assessment of general (physical and mental) health and quality of life [9, 12]. The instrument has been validated against the Nottingham Health Profile [13] and covers multiple health dimensions: physical functioning (10 items), emotional well‐being (5 items), social functioning (2 items), limitations due to physical health (4 items), emotional problems (3 items), vitality (energy/fatigue) (4 items), bodily pain (2 items), and perceived general health (5 items). A final item assesses the subject’s perceived health change dimension from the year prior. Each dimension was independently scored 1–100, according to the RAND, with higher scores denoting better quality of life. The scale was also validated against respiratory symptoms and pulmonary function in 34 patients with IPF and 34 matched normal subjects [14]. In patients with IPF, the scale’s minimally important difference ranges from 2 to 4 [15].

The University of California at San Diego SOBQ is a 24‐item, self‐reported tool used to assess dyspnea in daily activities. The SOBQ has been validated using a cohort of pulmonary rehabilitation patients against clinically established measures of dyspnea [10]. The first 21 items assess the severity of shortness of breath during common activities, while the last three items assess the limitations from and fear of shortness of breath and overexertion. Each item is rated on a six‐point scale ranging from 0 = *not at all* to 5 = *maximal or unable to do because of breathlessness*. Total scores range from 0 to 120, with higher scores indicating more severe dyspnea. The minimally important difference was established at 8 units [16].

For N‐GSSIQ, NIH PROMIS Forms, and UCSD SOBQ, total scores were calculated, and for SF‐36, individual domain scores were calculated. Higher scores on all symptom scales indicate higher symptom burden, whereas higher scores on the SF‐36 denote better quality of life.

### 2.4. Pulmonary Function Tests (PFT) and Gender, Age, and Pulmonary Physiology (GAP) Model

PFTs, including spirometry, plethysmography, and diffusing capacity of the lung for carbon monoxide corrected for hemoglobin (DLCOc), were performed using a Jaeger Masterscreen Body Box (Jaeger/CareFusion, Hoechberg, Germany) under standard procedures [17, 18]. Blood was drawn the morning after polysomnography (PSG) to measure hemoglobin level for the DLCO correction. A six‐minute walk test (6MWT) was conducted using the Masimo Radical‐7 (Masimo, Irvine, CA), following standard protocol [19]. Parameters from PFT used in the analyses include forced vital capacity (FVC), forced expiratory volume (FEV_1_), total lung capacity (TLC), and DLCOc, all as percentages of predicted values. From the 6MWT, we used maximum distance walked (ft.) and minimum SpO_2_ attained.

Using demographic and physiologic data, we calculated the gender, age, and two lung physiology variables [FVC% predicted and DLCOc %predicted]) score and stage (I–III), a validated multidimensional risk model, with higher scores and stage predicting worse clinical outcomes and prognosis [20].

### 2.5. PSG

PSG was performed over the subject’s night of habitual sleep employing the standard montage [21]: bilateral electrooculograms, electroencephalogram (F3‐M2, F4‐M1, C3‐M2, C4‐M1, O1‐M2, and O2‐M1), bipolar chin and anterior tibialis electromyograms, two electrocardiographic (ECG) standard leads, snore microphone, nasal and oral airflow thermocouples (MVAP Medical Services, Inc., Thousand Oaks, CA), nasal pressure cannula (Pro‐Tech Services, Inc., Woodinville, WA), thoracic and abdominal excursions by calibrated inductance plethysmography (Inductotrace, Ambulatory Monitoring, Inc., Ardsley, NY) with zRIP belts (Pro‐Tech Services, Inc., Mukilteo, WA), finger oximetry, and body position. For the first 13 subjects, PSG was recorded on a Grass Technologies system (Grass Technologies, TWin 4.5.3.23, West Warwick, RI), and for the remaining 11 subjects on a Compumedics Grael system with a Grael DC module (Compumedics, Profusion PSG V4.5 Build 574, Victoria, Australia). Subjects unable to sleep in the laboratory for at least 2 h on the first night were offered the opportunity to return for a repeat attempt at PSG.

Sleep stages (in 30 s epochs), respiratory events, leg movements (LMs), and arousals (respiratory, LM‐related, and spontaneous) were scored per standard American Academy of Sleep Medicine (AASM) criteria [21]. All studies were scored by a single technician (AP) and reviewed by an American Board of Internal Medicine and AASM‐certified Sleep Medicine physician (MT). Apnea was defined as a drop in peak airflow signal excursion by ≥ 90% of pre‐event baseline lasting ≥ 10 s. Apneas were scored as obstructive if they met all standard apnea criteria and were associated with continued or increased inspiratory effort throughout the entire period of absent airflow. Apneas were scored as central if they met all standard apnea criteria and were associated with absent inspiratory effort throughout the entire period of absent airflow. Apneas were defined as mixed if they met all standard apnea criteria and were associated with absent inspiratory effort in the initial portion of the event, followed by resumption of inspiratory effort in the second portion of the event. Hypopneas were defined as a drop in peak nasal pressure signal excursion by ≥ 30% of the pre‐event baseline, lasting ≥ 10 s, accompanied by a ≥ 3% oxygen desaturation and/or arousal. A respiratory effort‐related arousal (RERA) was defined as a sequence of breaths lasting ≥ 10 s characterized by either a drop in nasal pressure signal and/or an increase in respiratory effort leading to arousal when the sequence of breaths did not meet the criteria for an apnea or hypopnea.

A LM was scored by the presence of an 8 µV increase in electromyography voltage above baseline, a duration between 0.5 and 10 s, and the inception of the movement being > 0.5 s from both the onset and end of an apnea, hypopnea, or RERA. If ≥ 4 consecutive LMs occurred 5–90 s between the end and start of each LM, they were classified as periodic leg movements (PLMs). Otherwise, they were marked as isolated LM. Additionally, PLM arousals (PLMA and isolated leg movement arousal [LMA]) were scored as an arousal occurring within 0.5 s of the onset of a (periodic or isolated) LM.

The apnea–hypopnea index (AHI) was calculated as the total number of apneas and hypopneas divided by total hours of sleep. Respiratory disturbance index (RDI) was calculated as the total number of apneas, hypopneas, and RERAs divided by total hours of sleep. Respiratory arousal index (RAI) was calculated as the total number of respiratory event‐related arousals (apnea, hypopnea, and RERA‐related) divided by total hours of sleep. The isolated leg movement index (LMI) was calculated as the total number of isolated LMs divided by total hours of sleep, and the PLM index (PLMI) was calculated as the total number of PLMs divided by total hours of sleep. Isolated leg movement arousal index (LMAI) and periodic LMAI (PLMAI) were calculated as the number of isolated LMA and PLMA, respectively, per hour of sleep. Total arousal index (TAI) was calculated as the total number of arousals (spontaneous, respiratory, and all LM‐related) divided by total hours of sleep. Sleep efficiency was calculated as the time spent asleep divided by the time spent in bed from lights off to on, represented as a percentage.

From PSG, global sleep measures (TST, wake after sleep onset [WASO]), respiratory parameters (AHI, RDI, minimum oxygen saturation [MinSpO_2_], time with oxygen saturation under 88% as % of total sleep time [Time ≤ 88% as %TST]), LMs (isolated LMI and PLMI), and measures of sleep fragmentation (sleep efficiency, TAI, RAI, and isolated LMAI and PLMAI) were extracted for analysis.

### 2.6. Data Analysis

The GERD study variables consisted of (1) clinically established diagnoses extracted from EMR chart reviews and (2) nocturnal symptoms assessed with N‐GSSIQ.

### 2.7. Statistical Analysis

We used SAS software (SAS Institute, Version 9.4, Cary, NC) for analysis and R (R Core Team, Version 4.2.1, Vienna, Austria) and RStudio (RStudio, Build 576, Boston, MA) for graphical design. Continuous data were tested for normality using the Shapiro–Wilk test for normality in the SAS PROC UNIVARIATE, and, because most did not follow a normal distribution, data are summarized as medians along with 25% and 75% quartiles (Q1, Q3), or with the interquartile range in the figures. Categorical variables are presented as numbers (percentages). Spearman rank‐order nonparametric tests were used to assess correlations between continuous variables of interest. For two‐group comparisons, Wilcoxon rank sum (WRS) nonparametric tests were used for continuous variables and Fisher exact tests for categorical data, with two‐sided *p* values being reported. Analyses of AHI‐based OSA severity (mild: 5–14.9, moderate: 15–29.9, and severe: ≥ 30 events/h) employed generalized linear regression. *p* values < 0.05 indicated statistical significance.

## 3. Results

### 3.1. Baseline Characteristics

Among the 25 eligible subjects, one subject slept in the laboratory for only 48 min on the night of the PSG and was unwilling to return for a repeat attempt. Table 1 presents baseline characteristics of the remaining 24 subjects who completed the study. Overall, subjects were older, white males, overweight, with mild restriction and moderately impaired DLCOc, and a preponderance of comorbidities, similar to other reports [22]. Interestingly, 14 (58%) reported losing weight in the prior year. OSA had been previously clinically diagnosed in 9 (37%) subjects, among whom 4 (44%) were using PAP (in the prior 30 days, median [Q1, Q3] use was 7.4 [5.3, 7.8] h and 95.5% [71.5%, 99%] nights with use ≥ 4 h/night), and 4 (44%) others reported they had lost weight for OSA treatment.

**Table 1:** Baseline characteristics of *n* = 24 subjects with idiopathic pulmonary fibrosis.

	Median (Q1, Q3) or number (%)	Range
Age (y.o.)	71.5 (65.5, 74.5)	50.0–84.0
Sex (M/F)	17 (71%)/7 (29%)	—
Race		
White/Caucasian	23 (96%)	—
Other	1 (4%)	—
IPF duration of diagnosis (years)	2.0 (1.0, 4.0)	1–10.0
BMI (kg/m^2^)	27.0 (24.9, 30.3)	19.9–42.5
Neck circumference (inches)	15.1 (14.7, 16.3)	11.7–19.0
Weight (lbs.)	182.9 (161.5, 208.0)	123.3–324.7
Weight change/prior year		
Weight change (Y/N)	19 (79%)/5 (21%)	—
Weight loss (*n* = 14) (lbs.)	12.0 (7.0, 25.0)	3.5–50.0
Weight gain (*n* = 5) (lbs.)	5.0 (3.0, 20.0)	3.0–20.0
Smoking history	17 (71%)	—
Pack‐years	22.5 (14.0, 40.0)	2.0–50.0
Pulmonary and exercise function		
FVC (% predicted)	73.0 (63.5, 81.5)	41.0–120.0
FEV1 (% predicted)	84.0 (68.5, 90.5)	42.0–110.0
FEV1/FVC	82.0 (75.9, 84.1)	64.85–94.15
TLC (% predicted)	72.5 (64.5, 77.5)	50.0–117.0
DLCOc (% predicted)^∗^	49.5 (43.0, 60.5)	33.0–78.0
Hemoglobin (g/dL)	14.7 (13.8, 15.6)	11.1–17.5
Home O_2_ use (Y/N)	6 (25%)/18 (75%)	—
6MWT Min SpO2	84.0 (82.0, 88.0)	75.0–93.0
6MWT distance walked (feet)	1339.3 (1129.0, 1555.1)	587.0–2283.5
GAP measures		
GAP score	3.5 (3.0, 4.5)	2.0–6.0
GAP stage (1/2/3)	12 (50%)/9 (38%)/3 (13%)	—
Health care use due to IPF in the past 12 months		
ER or unscheduled clinic visits	0.0 (0.0, 0.0)	0.0–3.0
Hospitalizations	0.0 (0.0, 0.0)	0.0–0.0
Steroid bursts	0.0 (0.0, 0.0)	0.0–3.0
Health care use due to IPF ever		
Hospitalizations	0.0 (0.0, 0.0)	0 0.0–2.0
ICU admissions	0.0 (0.0, 0.0)	0.0–1.0
Mechanical ventilation	0.0 (0.0, 0.0)	0.0–0.0
Comorbidities		
Obstructive sleep apnea (OSA)	9 (44%)	
Using positive airway pressure (PAP)	4 (17%)	
Hypertension	10 (42%)	
Coronary artery disease	5 (21%)	
Congestive heart failure	1 (4%)	
Cerebral vascular accident	1 (4%)	
Hypercholesterolemia	13 (54%)	
Diabetes mellitus	5 (21%)	
Thyroid disease	3 (13%)	
Chronic kidney disease	0 (0%)	
Anemia	1 (4%)	
Psychiatric disorder	5 (21%)	

*Note:* Q1 and Q3 are 25% and 75% quartiles.

Abbreviations: BMI, body mass index; DLCOc, diffusing capacity of the lung for carbon monoxide corrected for hemoglobin; FEV_1_, forced expiratory volume in the first second of FVC maneuver; FVC, forced vital capacity; IPF, idiopathic pulmonary fibrosis; TLC, total lung capacity.

^∗^Data could be obtained in *n* = 20 subjects.

### 3.2. GERD, Daytime Symptoms, and Questionnaires

Seventeen (71%) subjects had a prior clinical GERD diagnosis, and 16 (94%) of them were on treatment (Table 2). N‐GSSIQ scores indicated a burden of nocturnal GERD, and although N‐GSSIQ scores were overall 3x higher in individuals with a GERD diagnosis versus those without, this was not statistically significant (1.5 [0.5, 5.0] vs. 0.5 [0, 3.0], *p* = 0.287). PAP use was not related to prior GERD diagnosis, as 3/17 (18%) patients with GERD and 1/7 (14%) of those without GERD (*p* = 1.0) were on PAP. Likewise, no difference in N‐GSSIQ was found between PAP users versus nonusers (3.5 [2.8, 4.5] vs. 0.5 [0, 5.0], *p* = 0.196).

**Table 2:** GERD measures, daytime symptoms, and questionnaire scores.

	Number (%) or median (Q1, Q3)
GERD measures:	
GERD dx (Y/N)	17 (71%)/7 (29%)
GERD medication (Y/N)	16 (67%)/8 (23%)
N‐GSSIQ score	1.3 (0.0, 5.0)
Daytime symptoms (Y/N):	
Problem with sleepiness	13 (54%)/11 (46%)
Problem with fatigue	16 (67%)/8 (33%)
Problem with lack of energy	18 (75%)/11 (25%)
Problem with tiredness	15 (63%)/9 (37%)
Questionnaires:	
PROMIS scales:	
Sleep impairment	46.4 (41.4, 54.6)
Sleep disturbance	49.1 (44.6, 53.4)
Fatigue	51.0 (41.9, 56.7)
Anxiety	47.8 (37.1, 51.3)
Depression	41.5 (38.2, 47.5)
SOBQ	28.0 (13.5, 49.0)
SF‐36 domains:	
Physical functioning	65.0 (40.0, 75.0)
Role limitations (physical)	25.0 (0.0, 100.0)
Role limitations (emotional)	100.0 (83.3, 100.0)
Energy/fatigue	55.0 (37.5, 72.5)
Emotional well‐being	88.0 (80.0, 92.0)
Social functioning	100.0 (68.8, 100.0)
Pain	77.5 (62.5, 100.0)
General health	40.0 (30.0, 55.0)
Health change	50.0 (25.0, 50.0)

*Note:* Q1 and Q3 are 25% and 75% quartiles.

Abbreviations: GERD, gastroesophageal reflux disease; N‐GSSIQ, Nocturnal Gastroesophageal Reflux Disease Symptom Severity and Impact Questionnaire; PROMIS, Patient‐Reported Outcomes Measurement Information System; SF‐36, Short Form‐6 Health Survey; SOBQ, Shortness of Breath Questionnaire.

Among daytime symptoms, the most often reported were lack of energy (18/24, 75%) and fatigue (16/24, 67%), and the least common was sleepiness (13/24, 54%) (Table 2). Questionnaires’ scores are also presented in Table 2.

### 3.3. Sleep Disturbance, OSA, and Severity

Table 3 presents the PSG parameters first for the whole group and then stratified by OSA (AHI ≥ 5/h) severity. Overall, as previously reported, the sleep architecture was marked by reduced sleep efficiency, increased wakefulness after sleep onset (WASO), increased light (N1 and N2), and reduced N3 and rapid eye movement (REM) sleep.

**Table 3:** Sleep parameters for the entire group and by obstructive sleep apnea severity.

	All subjects	No OSA (AHI < 5)	Mild OSA (AHI = 5–15)	Moderate OSA (AHI = 15–29.9)	Severe OSA (AHI ≥ 30)	Statistics by OSA severity	
	*n* = 24	*n* = 5 (21%)	*n* = 4 (17%)	*n* = 10 (42%)	*n* = 5 (21%)	*R* ^2^	*p* value
TST (min.)	323.5 (263.5, 367.3)	359.5 (323.0, 390.5)	352.5 (310.0, 381.8)	306.8 (264.5, 325.0)	257.0 (240.0, 262.0)	0.225	0.156
WASO (min.)	113.9 (92.0, 191.5)	96.5 (85.2, 120.8)	80.3 (52.3, 158.7)	122.8 (103.0, 182.0)	184.8 (181.0, 262.0)	0.215	0.175
Sleep efficiency (%)	69.1 (55.2, 79.4)	79.1 (74.4, 81.0)	79.0 (66.1, 84.9)	65.5 (56.3, 76.3)	52.5 (49.5, 58.6)	0.244	0.125
N1 (%TST)	14.4 (11.2, 19.1)	12.9 (10.0, 13.5)	12.0 (6.7, 14.3)	15.4 (11.6, 19.8)	21.2 (18.3, 23.5)	0.328	**0.043**
N2 (%TST)	65.8 (57.8, 75.5)	64.8 (57.6, 74.4)	73.9 (62.7, 85.8)	66.3 (54.6, 72.1)	58.9 (58.0, 67.3)	0.121	0.451
N3 (%TST)	10.2 (2.7, 18.4)	11.6 (9.2, 19.5)	10.8 (3.6, 15.9)	9.1 (2.8, 17.6)	13.0 (0.0, 20.8)	0.105	0.517
REM (%TST)	6.7 (3.5, 11.0)	9.0 (4.0, 12.4)	5.5 (2.8, 8.5)	8.5 (6.0, 13.4)	1.4 (0.0, 5.5)	0.234	0.141
Supine sleep time (%TST)	34.1 (10.1, 72.2)	22.5 (10.8, 47.1)	76.3 (31.0, 100.0)	17.3 (8.8, 32.0)	70.6 (63.7, 73,7)	0.292	0.070
AHI (events/h)	18.2 (8.1, 27.8)	1.7 (0.9, 3.8)	9.2 (8.1, 12.0)	21.9 (16.8, 24.0)	56.5 (37.1, 59.5)	0.705	**0.001**
Supine AHI (events/h) (*n* = 21)^∗^	26.5 (11.1, 52.3)	5.0 (3.2, 6.4)	12.9 (9.1, 15.5)	35.9 (25.6, 48.3)	67.7 (64.4, 85.5)	0.500	**0.003**
REM AHI (events/h) (*n* = 22)^∗^	30.6 (13.7, 45.0)	0.0 (0.0, 24.0)	19.3 (3.2, 56.2)	31.1 (20.4, 45.0)	52.2 (42.5, 68.6)	0.150	0.343
RDI (events/h)	29.9 (21.5, 43.2)	9.0 (5.0, 14.4)	21.5 (18.2, 30.5)	31.4 (29.3, 34.7)	65.9 (46.5, 77.3)	0.681	**0.001**
Supine RDI (events/h) (*n* = 21)^∗^	48.0 (35.4, 66.2)	29.0 (21.9, 34.3)	32.3 (22.3, 42.6)	51.2 (47.4, 59.3)	81.8 (78.1, 91.9)	0.391	**0.017**
REM RDI (events/h) (*n* = 22)^∗^	40.8 (15.8, 54.5)	10.5 (0.0, 44.6)	28.7 (12.1, 60.8)	39.8 (22.6, 53.7)	57.4 (47.5, 68.6)	0.082	0.628
Min. SpO2 (%)	86.0 (83.0, 89.0)	90.0 (88.0, 92.0)	89.0 (88.5, 89.5)	83.5 (82.0, 87.0)	83.0 (81.0, 83.6)	0.422	**0.011**
Time ≤ 88% (%TST)	0.3 (0.0, 2.4)	0.0 (0.0, 0.1)	0.0 (0.0, 0.1)	2.1 (0.1, 4.2)	1.5 (0.8, 15.2)	0.248	0.120
Isolated LMI (events/h)	6.5 (5.1, 11.5)	5.8 (4.8, 5.9)	11.3 (7.2, 14.8)	6.5 (5.4, 10.0)	11.0 (4.2, 11.9)	0.168	0.289
PLMI (events/h)	30.7 (10.7, 47.0)	43.8 (5.9, 52.1)	33.7 (30.7, 39.1)	32.5 (2.9, 50.1)	23.7 (15.4, 26.8)	0.028	0.901
RAI (arousals/h)	23.1 (14.2, 37.9)	8.4 (5.5, 15.1)	14.3 (7.8, 26.2)	23.1 (17.2, 31.0)	56.3 (41.1, 56.9)	0.548	**0.001**
Isolated LMAI (arousals/h)	1.3 (0.9, 2.1)	0.9 (0.8, 1.0)	1.1 (0.6, 1.7)	1.5 (1.1, 2.1)	2.5 (1.3, 2.7)	0.167	0.292
PLMAI (arousals/h)	3.7 (2.0, 5.6)	11.8 (3.3, 14.2)	4.7 (3.2, 5.4)	2.2 (0.6, 5.3)	2.6 (2.3, 5.5)	0.239	0.133
SAI (arousals/h)	5.0 (3.4, 9.2)	7.8 (2.3, 9.9)	5.6 (3.0, 8.1)	4.1 (3.4, 5.6)	11.9 (6.4, 12.0)	0.176	0.266
TAI (arousals/h)	35.3 (24.2, 51.2)	31.0 (23.5, 31.0)	26.3 (15.2, 40.7)	35.3 (24.8, 37.0)	76.2 (56.5, 83.8)	0.504	**0.003**

*Note:* Data shown as median (Q1 and Q3 are 25% and 75% quartiles). N1‐3‐non‐rapid eye movement sleep stages; time ≤ 88% as %TST‐time with oxygen saturation under 88% as a percentage of total sleep time; boldface noted for significant associations (*p* < 0.05).

Abbreviations: AHI, apnea–hypopnea index; LMAI, isolated leg movement arousal index; LMI, isolated leg movement index; Min. SpO2, minimum oxygen saturation recorded during sleep; OSA, obstructive sleep apnea; PLMAI, periodic leg movement arousal index; PLMI, periodic leg movement index; RAI, respiratory arousal index; RDI, respiratory disturbance index; REM, rapid eye movement sleep stage; SAI, spontaneous arousal index; TAI, total arousal index (spontaneous, respiratory, and all leg movement‐related); TST, total sleep time; WASO, wake after sleep onset.

^∗^Reported for subjects who achieved supine or REM sleep.

Secondly, we found a substantial amount of SDB, with overall AHI across the entire cohort reaching moderate OSA severity (18.2 [8.1, 27.8]/h) and RDI nearly in the severe range (29.9 [21.5, 43.2]/h). When defining OSA by AHI ≥ 5/h, 19 (79%) subjects met the diagnosis with an overall AHI in the moderate range (23.8 [15.7, 32.5]/h), and with most subjects, 15/19 (79%), having moderate or severe disease (Table 3). Regarding prior clinical recognition, 11/19 (58%) of OSA (AHI ≥ 5/h) cases on the study PSG have not been clinically diagnosed, of which 4/11 (36%) were in the mild, 5/11 (45%) moderate and 2/11 (18%) severe OSA categories. One subject with previously diagnosed OSA did not have the disease on the study PSG. When defining OSA by RDI ≥ 5/h, the disease was present in almost all subjects (23/24, 96%) and was overall severe (RDI 30.2 [23.8, 45.1]/h); the one subject without OSA by this criterion had not been clinically diagnosed with OSA either.

PLMs were also very common, with an overall PLMI of 30.7 (10.7, 47.0)/h, exceeding 5/h in 19 (79%) and 25/h in 16 (67%) subjects. However, only a few PLMs were associated with detectable arousals (PLMAI 3.7 [2.0, 5.6]/h).

With increased OSA severity, apart from the expected changes in respiratory parameters and increases in related arousals, generally, no significant differences were noted in the sleep architecture or LMs (Table 3).

### 3.4. Associations of GERD With Daytime Symptoms and Questionnaires

A GERD diagnosis did not relate to daytime symptoms (sleepiness, fatigue, lack of energy, and tiredness) (all *p* values > 0.10). However, N‐GSSIQ scores were significantly higher with reported fatigue and tiredness (both *p* < 0.05), and trends were noted with daytime sleepiness (*p* = 0.085) and lack of energy (*p* = 0.054) (Figure S1).

Associations of GERD measures with questionnaires are shown in Table 4. While GERD diagnosis was not associated with any, N‐GSSIQ scores related to multiple scales. Notably, increasing N‐GSSIQ score significantly correlated with increased PROMIS Fatigue (*p* = 0.001) and Sleep Impairment (reflective of daytime sleepiness) (*p* = 0.010), and increased SOBQ score (*p* = 0.031). Like the relationships with daytime symptoms, higher N‐GSSIQ correlated with lower scores on SF‐36 Energy/Fatigue, Pain, Social Functioning and Health Change in the prior year (all *p* < 0.05).

**Table 4:** Associations of GERD measures with questionnaire scores.

	GERD diagnosis			N‐GSSIQ score	
	No (*n* = 7)	Yes (*n* = 17)	*p* value	Rho	*p* value
	Median (Q1, Q3)	Median (Q1, Q3)			
PROMIS scales:					
Sleep Impairment	47.3 (41.4, 48.9)	45.5 (38.7, 55.1)	0.975	0.518	**0.010**
Sleep Disturbance	45.3 (43.9, 51.3)	50.2 (46.7, 54.3)	0.227	0.347	0.097
Fatigue	51.5 (41.0, 57.5)	50.4 (42.8, 55.6)	0.801	0.622	**0.001**
Anxiety	37.1 (37.1, 47.8)	47.8 (37.1, 53.2)	0.345	0.248	0.242
Depression	44.7 (38.2, 47.5)	38.2 (38.2, 47.5)	0.946	0.152	0.479
SOBQ	21.0 (13.0, 39.0)	29.0 (14.0, 49.0)	0.638	0.441	**0.031**
SF‐36 domains:					
Physical Functioning	65.0 (35.0, 80.0)	65.0 (45.0, 65.0)	0.975	−0.295	0.162
Role Limitations (Physical)	25.0 (0.0, 100.0)	25.0 (0.0, 75.0)	0.795	−0.162	0.450
Role Limitations (Emotional)	100.0 (100.0, 100.0)	100.0 (66.7, 100.0)	0.459	−0.059	0.785
Energy/fatigue	60.0 (40.0, 80.0)	55.0 (35.0, 65.0)	0.416	−0.491	**0.015**
Emotional Well‐being	88.0 (84.0, 92.0)	84.0 (80.0, 92.0)	0.245	−0.279	0.187
Social Functioning	100.0 (75.0, 100.0)	100.0 (62.5, 100.0)	0.681	−0.556	**0.005**
Pain	77.5 (57.5, 80.0)	70.0 (67.5, 100.0)	0.848	−0.444	**0.030**
General Health	55.0 (40.0, 65.0)	40.0 (30.0, 55.0)	0.081	−0.297	0.159
Health Change in the Past Year	50.0 (25.0, 75.0)	50.0 (25.0, 50.0)	0.177	−0.441	**0.035**

*Note:* Boldface is noted for significant associations (*p* < 0.05). Q1 and Q3 are 25% and 75% quartiles; N1‐3‐non‐rapid eye movement sleep stages; Rho, correlation coefficient from Spearman rank‐order nonparametric test.

Abbreviations: GERD, gastroesophageal reflux disease; LMAI, isolated leg movement arousal index; LMI, isolated leg movement index; N‐GSSIQ, Nocturnal Gastroesophageal Reflux Disease Symptom Severity and Impact Questionnaire; PLMAI, periodic leg movement arousal index; PLMI, periodic leg movement index; PSG, polysomnography; REM, rapid eye movement sleep stage; SAI, spontaneous arousal index; TAI, total arousal index; TST, total sleep time; WASO, wake after sleep onset.

### 3.5. Relationships of GERD With Sleep Disruption on PSG

As shown in Table 5, a GERD diagnosis was significantly associated with increased N2 (*p* = 0.047), reduced REM sleep (*p* = 0.039) and increased PLMI (*p* = 0.047). Likewise, as N‐GSSIQ score increased, PLMI significantly increased (*p* = 0.023) (Figure 1(a), Table 5). Although subjects with GERD had a higher PLMAI, this was not statistically significant (Table 5). Also, no association of N‐GSSIQ score with PLMAI was noted (Figure 1(b)). Time sleeping supine (as %TST), a position known to facilitate reflux and extend the refluxate contact time with the tissues, was not related to GERD diagnosis (*p* = 0.408) or N‐GSSIQ score (*p* = 0.436) (Table 5).

**Table 5:** Potential role of GERD in the sleep disruption on nocturnal PSG.

	GERD dx			N‐GSSIQ score	
	No (*n* = 7)	Yes (*n* = 17)	*p* value		
	Median (Q1, Q3)	Median (Q1, Q3)		Rho	*p* value
TST (min.)	289.5 (264.5, 375.0)	324.0 (262.0, 359.5)	0.638	0.185	0.387
WASO (min.)	160.2 (85.2, 182.0)	107.0 (94.5, 198.1)	0.975	0.134	0.531
Sleep efficiency (%)	62.2 (52.5, 79.1)	69.3 (56.3, 79.7)	0.900	−0.018	0.933
N1 (%TST)	15.0 (11.6, 19.8)	13.5 (10.8, 18.3)	0.684	−0.087	0.685
N2 (%TST)	52.2 (30.6, 72.1)	66.6 (60.6, 76.5)	**0.047**	0.172	0.422
N3 (%TST)	8.6 (2.8, 19.5)	10.7 (2.6, 17.4)	0.950	0.094	0.663
REM (%TST)	10.0 (7.1, 13.4)	5.5 (1.4, 9.9)	**0.039**	−0.193	0.367
Supine sleep time (%TST)	32.8 (0.0, 70.6)	35.4 (12.0, 73.7)	0.408	−0.167	0.436
Isolated LMI (events/h)	5.8 (4.8, 7.2)	9.1 (5.3, 11.9)	0.492	0.110	0.608
PLMI (events/h)	5.9 (2.9, 29.5)	35.6 (29.6, 52.1)	**0.047**	0.462	**0.023**
Isolated LMAI (arousals/h)	1.1 (0.3, 2.5)	1.3 (0.9, 2.0)	0.802	−0.074	0.730
PLMAI (arousals/h)	2.5 (0.9, 5.33)	4.3 (2.3, 11.8)	0.206	0.237	0.266
SAI (arousals/h)	7.8 (3.5, 17.7)	4.1 (3.1, 7.7)	0.083	−0.210	0.325
TAI (arousals/h)	35.5 (30.9, 74.9)	35.0 (23.5, 50.6)	0.730	−0.039	0.858

*Note:* Boldface is noted for significant associations (*p* < 0.05). Q1 and Q3 are 25% and 75% quartiles; Rho, correlation coefficient from Spearman rank‐order nonparametric test; N1‐3, non‐rapid eye movement sleep stages.

Abbreviations: GERD, gastroesophageal reflux disease; LMAI, isolated leg movement arousal index; LMI, isolated leg movement index; N‐GSSIQ, Nocturnal Gastroesophageal Reflux Disease Symptom Severity and Impact Questionnaire; PLMAI, periodic leg movement arousal index; PLMI, periodic leg movement index; PSG, polysomnography; REM, rapid eye movement sleep stage; SAI, spontaneous arousal index; TAI, total arousal index; TST, total sleep time; WASO, wake after sleep onset.

Figure 1Relationships of N‐GSSIQ scores with PLM on PSG. Higher N‐GSSIQ correlated with higher PLMI (a) (*p* = 0.023). No significant association was found with PLMAI (b). Abbreviations: N‐GSSIQ, Nocturnal Gastroesophageal Reflux Disease Symptom Severity and Impact Questionnaire; PSG, polysomnography; PLM, periodic leg movements; PLMI, periodic leg movements index; PLMAI, periodic leg movement arousal index; *Rho,* correlation coefficient from Spearman rank‐order nonparametric test.

**Figure:**
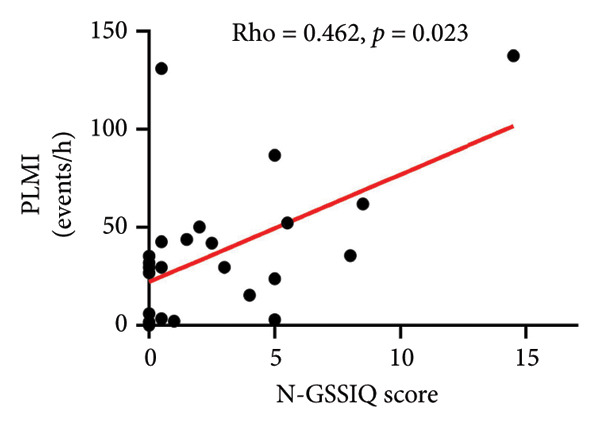
(a)

**Figure:**
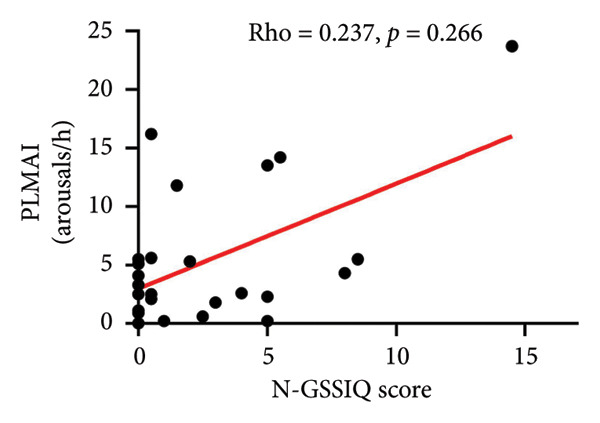
(b)

### 3.6. Associations of GERD and PSG Sleep Disturbance With PFT, 6MWT, GAP Measures, and IPF‐Related Healthcare Burden

Neither a GERD diagnosis (Figure S2) nor an N‐GSSIQ score was significantly related to PFTs, 6MWT, and GAP measures (all *p* > 0.10) (Table 6); likewise, no associations with prior 12‐month IPF‐related hospitalizations, unscheduled doctor visits, and steroid bursts or with lifetime hospitalizations, ICU admissions, and the need for mechanical ventilation were found (all *p* > 0.05, data not shown).

**Table 6:** Associations of N‐GSSIQ score and nocturnal PSG sleep parameters with pulmonary and exercise function.

	FVC (% predicted)		FEV1 (% predicted)		FEV1/FVC		TLC (% predicted)		DLCOc (% predicted)^∗^		Distance walked (feet)		GAP score (points)	
	Rho	*p* value	Rho	*p* value	Rho	*p* value	Rho	*p* value	Rho	*p* value	Rho	*p* value	Rho	*p* value
GERD:														
GERD diagnosis (Y/N)	—	0.552	—	0.777	—	0.126	—	0.472	—	0.699	—	0.802	—	0.135
N‐GSSIQ score	0.053	0.804	0.048	0.823	−0.019	0.932	−0.082	0.702	0.013	0.957	0.076	0.724	0.220	0.300
PSG/global sleep:														
TST (min.)	0.682	**0.001**	0.612	**0.002**	−0.192	0.368	0.587	**0.003**	0.300	0.199	0.480	**0.018**	−0.512	**0.011**
WASO (min.)	−0.592	**0.002**	−0.517	**0.010**	0.149	0.488	−0.573	**0.003**	−0.466	**0.039**	−0.384	0.064	0.530	**0.008**
Sleep efficiency (%)	0.604	**0.002**	0.532	**0.008**	−0.170	0.426	0.524	**0.009**	0.357	0.122	0.365	0.080	−0.462	**0.023**
PSG/SDB metrics:														
AHI (events/h)	−0.022	0.918	0.168	0.432	0.015	0.945	0.069	0.749	−0.439	0.053	−0.299	0.156	0.097	0.653
Supine AHI (events/h)	0.002	0.994	0.134	0.532	−0.005	0.981	0.108	0.615	−0.416	0.068	−0.388	0.061	0.187	0.416
NREM AHI (events/h)	−0.008	0.969	0.163	0.445	−0.006	0.978	0.083	0.699	−0.432	0.057	−0.355	0.088	0.124	0.563
REM AHI (events/h)	0.206	0.333	0.244	0.251	0.035	0.871	0.201	0.347	−0.029	0.904	0.138	0.519	−0.381	0.080
RDI (events/h)	−0.175	0.413	0.019	0.931	0.142	0.509	−0.075	0.728	−0.609	**0.004**	−0.354	0.090	0.095	0.660
Supine RDI (events/h)	−0.088	0.681	0.047	0.826	0.094	0.662	−0.047	0.829	−0.487	**0.029**	−0.345	0.099	0.230	0.315
NREM RDI (events/h)	−0.169	0.429	0.017	0.936	0.123	0.568	−0.080	0.710	−0.579	**0.008**	−0.400	0.053	0.154	0.472
REM RDI (events/h)	0.249	0.241	0.290	0.170	0.116	0.588	0.244	0.251	−0.023	0.922	0.130	0.545	−0.463	**0.030**
Min. SpO2 (%)	0.116	0.588	−0.030	0.890	−0.235	0.269	0.158	0.460	0.350	0.130	0.118	0.584	0.027	0.901
Time ≤ 88% (%TST)	−0.139	0.517	−0.005	0.983	0.291	0.167	−0.127	0.554	−0.417	0.068	−0.152	0.478	−0.005	0.983
PSG/limb movements:														
PLMI (events/h)	0.097	0.653	0.047	0.829	−0.100	0.642	−0.030	0.889	0.177	0.456	−0.054	0.802	−0.091	0.674
PSG/Sleep fragmentation:														
RAI (arousals/h)	−0.206	0.335	−0.021	0.921	0.201	0.347	−0.126	0.557	−0.505	**0.023**	−0.336	0.109	0.167	0.435
PLMAI (arousals/h)	0.025	0.908	−0.004	0.986	−0.103	0.633	−0.035	0.872	0.108	0.649	−0.340	0.104	0.033	0.878
SAI (arousals/h)	−0.262	0.216	−0.173	0.420	0.334	0.110	−0.140	0.514	−0.460	**0.042**	−0.359	0.085	0.283	0.180
TAI (arousals/h)	−0.302	0.151	−0.176	0.411	0.137	0.523	−0.198	0.353	−0.484	**0.031**	−0.438	**0.033**	0.190	0.373

*Note:* Boldface is used for significant associations (*p* < 0.05). Rho, correlation coefficient from Spearman rank‐order nonparametric test; Time ≤ 88% as %TST‐time with oxygen saturation under 88% as a percentage of total sleep time.

Abbreviations: AHI, apnea–hypopnea index; DLCOc, diffusing capacity of the lung for carbon monoxide corrected for hemoglobin; FEV_1_, forced expiratory volume in the first second of FVC maneuver; FVC, forced vital capacity; GAP, gender, age, pulmonary physiology; GERD, gastroesophageal reflux disease; Min. SpO2, minimum oxygen saturation recorded during sleep; N‐GSSIQ, Nocturnal Gastroesophageal Reflux Disease Symptom Severity and Impact Questionnaire; NREM, non‐rapid eye movement sleep stages; PLMAI, periodic leg movement arousal index; PLMI, periodic leg movement index; PSG, polysomnography; RAI, respiratory arousal index; REM, rapid eye movement sleep stage; RDI, respiratory disturbance index; SAI, spontaneous arousal index; TAI, total arousal index; TLC, total lung capacity; TST, total sleep time; WASO, wake after sleep onset.

^∗^Data available in *n* = 20 subjects.

Relationships between PSG sleep disturbance and SDB with PFT and 6MWT variables are also shown in Table 6. Worse overall sleep (lower TST and efficiency and higher WASO) correlated with worse lung function (FVC%, FEV1%, TLC%, and DLCOc%), shorter distance walked, and higher GAP scores. Among SDB indices, higher RDI (Figure 2(a)), particularly during NREM sleep and supine position (Table 6), significantly correlated with worse DLCOc% predicted and shorter walked distance; similar trends were noted for AHI with these measures. However, no significant associations were found between PSG metrics and MinSpO_2_ on 6MWT (all *p* > 0.10). Generally, SDB indices did not significantly relate to GAP measures, except for REM RDI, which negatively related to GAP score (Table 6) and stage (rho = −0.480, *p* = 0.024) but not with any individual physiologic components of GAP (Table 6). Concerning associations of LMs, PLMI did not relate to any physiologic or GAP outcomes (Table 6). More sleep fragmentation (higher total arousal indices [TAI]), particularly related to respiratory events (RAI, Figure 2(b)) and spontaneous arousals (SAI), significantly correlated with lower DLCOc% and shorter distance walked (TAI only), but not with GAP measures (Table 6). Last, none of the above PSG measures related to the prior 12‐month and lifetime IPF‐related healthcare use measures presented in Table 1 (all *p* > 0.10, data not shown).

Figure 2Associations of sleep‐disordered breathing on PSG with DLCOc. Higher RDI (a) and RAI (b) significantly correlated with worse DLCOc. Footnote: ^a^Data available in *N* = 20 subjects. Abbreviations: PSG, polysomnography; DLCOc, diffusing capacity of the lung for carbon monoxide corrected for hemoglobin, as percent of predicted values; *Rho*, correlation coefficient from Spearman rank‐order nonparametric test.

**Figure:**
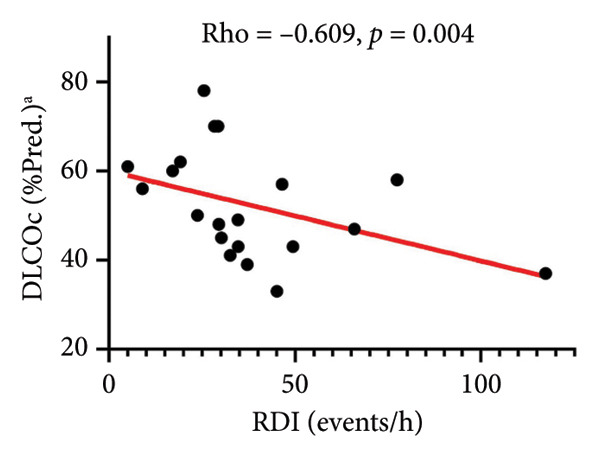
(a)

**Figure:**
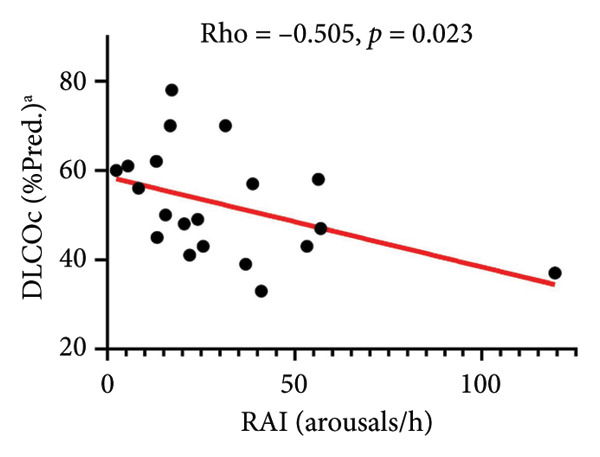
(b)

## 4. Discussion

### 4.1. Overview of Key Findings

Because GERD and OSA have each been recognized as potential contributors to adverse consequences in IPF [4, 23, 24], we tested the concurrent contributions of nocturnal GERD and SDB on physiologic and patient‐centered outcomes in IPF. We made several, including some novel, observations: (1) GERD is highly prevalent (17/24, 71%) with a high rate of medical treatment (16/17, 94%), yet a nocturnal burden was detected by tools not currently used in clinical practice; (2) this nocturnal GERD burden adversely related to the daytime complaints commonly reported by our subjects (Table 4, Figure S1); (3) the nocturnal contribution of GERD may relate to events leading to SAI (possibly pain) and PLMs (Table 5, Figure 1) that may go overlooked without laboratory‐based PSG testing; 4) GERD measures did not relate with daytime pulmonary and exercise physiology (Figure S2, Table 6); and (5) metrics of SDB obtainable solely through PSG testing, specifically RDI and RAI, correlated with worse DLCOc and walked distance (Figure 2, Table 6). These data emphasize the potential impact of unrecognized nocturnal GERD and associated sleep disruption on daytime symptoms that matter to patients; additionally, that SDB may worsen pulmonary physiology and accelerate IPF progression.

### 4.2. GERD and Its Impact on Patient‐Centered Outcomes

Known to be a common comorbidity of IPF, GERD was frequently diagnosed and treated in our population. However, when using a specific scale, we found a burden of nocturnal GERD, which was adversely related to daytime symptoms and quality of life. It is well known that individuals with nighttime heartburn often report sleep disturbance and excessive daytime sleepiness [25, 26]. Compared to wakefulness, during sleep there is prolonged clearance of esophageal acid, which produces an enhanced arousal response and increased swallowing frequency [27, 28]. On the other hand, relative to placebo, treatment with esomeprazole in patients with frequent nighttime heartburn and sleep disturbance leads to significant resolution of heartburn and reflux‐associated sleep complaints as well as improvement in sleep quality [29]. In this context, our findings may have important implications for clinical practice. This is because, in multiple prior IPF studies where GERD and OSA were not concurrently assessed, OSA metrics did not relate to daytime symptoms [22, 30], suggesting that other factors may contribute to daytime complaints. Second, the current IPF clinical guidelines [31] neither recommend assessing nocturnal GERD, nor any specific instruments for this purpose, like the one utilized in this study. Our results emphasize a need to refine current GERD monitoring by incorporating nocturnal control using multidimensional instruments that capture aspects most relevant to patients.

### 4.3. Possible Mechanisms of Nocturnal GERD‐Related Daytime Dysfunction

Our data suggest two possible pathways whereby nocturnal GERD contributes to daytime complaints. First, nocturnal GERD could induce pain and disrupt sleep, as GERD metrics adversely related to the SF‐36 pain domain (N‐GSSIQ score, Table 4) and PSG measures of sleep disruption (Table 5). Second, we observed a high burden of PLMs, larger than reported in one earlier study in IPF [32], and for the first time, we report significant associations of both GERD metrics used with higher PLMI (Table 5, Figure 1(a)). These observations suggest that GERD may either play a direct pathophysiologic role and/or be a marker of unassessed contributing factors to PLM. That is, nocturnal GERD‐related discomfort (heartburn, pain, etc.) may trigger leg kicks during sleep, which may or may not be associated with arousals detectable with the current scoring methodology. Additionally, GERD may be a marker of deficiency in iron stores, which are essential for the brain dopamine system function and PLM occurrence [33]. Even though hemoglobin levels did not show anemia (Table 1), iron deficiency may still be at play. We did not evaluate iron status, nor are there any reports on this matter in IPF patients. However, most of our subjects were on (likely long‐term) GERD therapy. Interestingly, prolonged PPI and H_2_‐blockade reduce dietary iron absorption [34–36], and the use of these medications is associated with restless legs syndrome in the general population [34]. These findings uncover a broad impact of GERD and its treatment on sleep disruption and daytime complaints, with important implications for patients, warranting further investigations.

### 4.4. Lack of GERD Associations With Pulmonary Physiology and GAP Metrics

We found no significant associations between GERD variables and outcomes on PFT, 6MWT, and GAP assessments. This aligns with previous reports in IPF patients showing no significant relationships between pH probe‐diagnosed GERD and FVC% or DLCO% predicted [37], which are also the physiologic constituents of the GAP model. The high recognition and medical treatment of GERD in our population likely diminished the overall impact of GERD on the physiology assessed. Indeed, acid suppression therapy in patients with IPF reduces the acid reflux events but increases the frequency of non‐acid reflux episodes, without improvement in respiratory symptoms [38]. Additionally, non‐acid reflux is more often linked to reflux hypersensitivity and non‐erosive reflux disease, whereas acid reflux more often causes erosive esophagitis, which is associated with pulmonary fibrosis [2, 39, 40]. Whether the residual burden of nocturnal GERD identified in our cohort relates to non‐acid reflux and whether such reflux produces more subliminal symptoms and sleep disruption than alterations is pulmonary physiology remains to be tested in future studies. Notwithstanding, our results suggest that nocturnal GERD may not substantially contribute to these physiologic outcomes and emphasize that increased value should also be placed on addressing other potentially impactful comorbidities, such as OSA.

### 4.5. Associations of SDB With Physiology

We observed a high burden of SDB (overall AHI: 18.2 [8.1, 27.8]/h) and OSA (AHI ≥ 5/h in 19/24, 79%), with the majority of cases (15/19, 79%) ranking in the moderate or severe OSA category (Table 3), known to have significant adverse health consequences [5, 41]. Yet, the disease was largely underdiagnosed, as over half of the OSA cases (11/19, 53%) had not been clinically recognized, with most (7/11, 64%) demonstrating moderate or severe disease on the study PSG. Moreover, it is important to note this high OSA burden occurred despite prior year substantial weight loss in most subjects and, overall, an overweight, not obese, body habitus (Table 1). This OSA burden parallels that reported from Europe (78%–82%) in patients with body habitus (BMI mean 26.3–27.3 kg/m^2^) [42, 43] similar to ours (mean ± standard deviation 28.0 ± 5.4 kg/m^2^), and lower than in an earlier, preantifibrotics era, U.S. sample (88%) but with a larger body habitus (mean 32.3 kg/m^2^) [30].

For the first time, we report significant associations of SDB severity, measured by RDI and RAI, with worse DLCO corrected for hemoglobin %predicted (Figure 2)—the gold standard for assessing pulmonary gas exchange on PFT—and trends for lower distance walked, but no associations with lung mechanics or volumes, or with GAP metrics (Table 6). Other studies in IPF patients that relied on DLCO uncorrected for hemoglobin did not find significant relationships of this measure with OSA [22, 30, 44], indicating the importance of using the most precise physiologic assessments when evaluating the potential impact of SDB on IPF. Additionally, our data suggest that such individual measures may be more relevant outcomes than composite scores. While IPF affects predominantly older men, OSA peaks in middle‐aged men and thus could precede and evolve alongside IPF, worsening its outcomes, as the literature also suggests [4]. Multiple underlying mechanisms may be at play in this relationship. Among them, OSA may accelerate progression of IPF‐related pulmonary hypertension, which has been associated with increased risk of mortality [45]. OSA, particularly through its hallmark feature, chronic intermittent hypoxia, could adversely impact cardiovascular health and risk for acute ischemic coronary events [5, 46] in these patients. Additionally, the recurrent obstructive events–imposed resistive breathing and associated desaturation‐reoxygenation episodes could promote distal airway epithelial injury and tissue inflammation, propagate the fibrotic parenchymal process and associated physiologic deficits, and lead to pulmonary capillary remodeling [47–50]—all features of IPF pathology.

### 4.6. Study Limitations and Strengths

The first limitation of our study arises from its descriptive, cross‐sectional design, limiting the ability to establish causality for the identified relationships, which could be bidirectional. While OSA may negatively impact IPF outcomes as our study suggests, conversely, IPF may contribute to SDB progression and evolution to OSA through multiple pathways. Among them, the reduction in lung volumes, exacerbated during REM sleep, could diminish the pharyngeal airway stiffness, as suggested by the negative correlations between wakefulness lung mechanics with AHI and REM AHI reported by Mermigkis et al. [32]. IPF exacerbations require treatment with high doses of corticosteroids, which could lead to fat redistribution to the neck area [4, 51] and alter the coordination of pharyngeal muscle groups responsible for upper airway stability [52]. Last, in preclinical models, we and others have found that airway and lung inflammation augment the ventilatory responses to hypoxia, thereby the controller loop gain, a precursor of breathing instability during sleep and apneas [53–55]. A second limitation of the study is that subject recruitment was adversely affected by the COVID‐19 pandemic, severely limiting enrollment in the study. Notwithstanding, even small, our sample’s characteristics parallel those reported in numerous other studies in the IPF population. Third, we lacked detailed objective characterization of GERD. The N‐GSSIQ provides a subjective, not an objective, assessment of the nocturnal GERD burden; additionally, data on the initial GERD diagnostic work‐up, which would have allowed us to discriminate among the various types of gastric refluxate (acid [pH < 4], weakly acid [pH 4–6.9], or non‐acid [pH ≥ 7]; liquid, gaseous, or mixed) and the extent of reflux syndromes (e.g., erosive esophagitis, Barrett’s, nonerosive reflux disease, reflux hypersensitivity, functional heartburn, or extraesophageal laryngopharyngeal reflux [LPR]), were not available. This information could have provided valuable insights into the pathophysiologic links between GERD and IPF. It is possible that different GERD phenotypes may trigger distinct mechanisms leading to chronic microaspiration, ultimately contributing to fibrotic remodeling of pulmonary parenchyma [2]. Gastric juice is a complex mixture of gastric acid, enzymes such as pepsin, bile acids, and pancreatic enzymes from the duodenum. These constituents can interact with each other and influence the biochemical activity and epithelial cellular toxicity of the refluxate, rendering it injurious even at neutral pH [38, 56, 57]. Others, such as LPR, more often caused by gaseous refluxate, may predispose to upper airway dysfunction, precipitating dysphagia with aspiration of nongastric material (saliva, food particles), particularly during the night, and OSA, which in turn could worsen IPF. Indeed, among patients with OSA, inflammatory changes in the hypopharynx and larynx consistent with those described in the LPR are prevalent, relate to apnea severity, and are associated with laryngeal sensory impairment and attenuation of the protective laryngeal adductor reflex [58, 59]. Moreover, LPR is common among patients with IPF, who demonstrate more instances of acidic LPR in the supine position and basic pH LPR while upright, compared to control subjects [60]. Collectively, these observations highlight important interactions of gastroesophageal reflux with upper and lower respiratory tract disorders through a “united airway” that may act as another link in the GERD–IPF relationship. We also lacked knowledge on host defenses against aspiration (e.g., glottic and epiglottic closures, cough and swallowing reflexes, peristalsis, and arousals from sleep) that act in concert to seal off the airway when foreign material is detected in the esophagus and hypopharynx; thus, we could not determine whether they were preserved, hyperactive, or deficient in our patients. The mixed results reported on the potential contribution of GERD to heightened cough reflex in IPF, corroborated with the lack of reduction in cough frequency with acid suppression, suggest that at least this protective mechanism may be intact in IPF patients. Additionally, in the study by Su et al., no differences in the laryngopharyngeal pH at the start of the cough relative to the background pH were noted, indicating no association of cough with LPR in IPF patients [60]. Further studies focused on the protective responses against pulmonary aspiration of gastroesophageal material are necessary. As we utilized a complex nighttime setup via PSG, adding a multiprobe combined impedance‐pH catheter for simultaneous pharyngeal and esophageal recordings, and objective assessment of the aforementioned reflux phenotypes would have been difficult to accept by our subjects and reduce the study’s feasibility, but this needs to be considered in future studies. Finally, we lacked objective information on adherence to antifibrotic and GERD medications, which may have impacted the relationships assessed. Nonetheless, we included an enrollment requirement for 6‐month stability in antifibrotic dosing, purposefully to ensure sufficient time for clinical drug monitoring, including adherence, through their providers. Additionally, subjects were asked to bring to the study visit all of their medications in the original bottles so we could verify their usage. Despite these shortcomings, owing to their clinical relevance, these initial exploratory results inform hypotheses and potential outcome selection for larger prospective interventional studies that could incorporate strategies addressing the limitations discussed.

Our study strengths stem from the detailed subjects’ characterization with objective, gold‐standard respiratory and sleep physiology, along with validated questionnaire‐based assessment of outcomes that matter to patients. Additionally, by simultaneously capturing data surrounding two of the most common IPF comorbidities occurring during sleep—nocturnal GERD and OSA—we provide insight into the concurrent contributions of each of these diseases to IPF outcomes.

## 5. Conclusions

In summary, while GERD is widely recognized in patients with IPF, a nocturnal GERD burden exists that is detectable on tools not currently included in clinical guidelines. This was associated with sleep disruption, daytime complaints, functional limitations, and poor quality of life. Underlying reasons for GERD‐related sleep disruption may relate to precipitating arousals and PLMs through nocturnal discomfort and/or effects of GERD treatments on iron absorption. More comprehensive GERD tools would be necessary in clinical practice to help abate the burden of sleep disruption and daytime complaints in IPF patients. Second, SDB and OSA are very common and adversely related to DLCOc and distance walked. Despite growing awareness of OSA’s health consequences, over half of our IPF patients had unrecognized and therefore untreated disease of a severity known to carry significant adverse health consequences. As others have called for [4], the expanding body of work on this topic reframes OSA as a significant opportunity for early intervention in IPF, offering the potential to slow the progression of this relentlessly progressive disease with a grim prognosis. In this framework, it is crucial to emphasize the need for (1) increased awareness of the link between OSA and IPF by education of both healthcare providers and patients; (2) routine screening for OSA in individuals with IPF or those at high risk of developing it; and (3) proactive management of OSA through personalized, precision‐informed multimodal approaches (e.g., lifestyle changes, PAP, dental devices, medications, and surgeries), given that no single, one‐size‐fits‐all treatment is universally effective for OSA. Our findings lay the groundwork for future, larger confirmatory studies, which should be multicenter in design to address the limitations discussed.

**Nomenclature**
6MWT: Six‐minute walk test
AASM: American Academy of Sleep Medicine
AHI: Apnea–hypopnea index
ATS: American Thoracic Society
BMI: Body mass index
CAD: Coronary artery disease
CHF: Congestive heart failure
COPD: Chronic obstructive pulmonary disease
CVA: Cardiovascular accident
DLCO: Diffusing capacity of the lung for carbon monoxide
DLCOc: Diffusing capacity of the lung for carbon monoxide, corrected for hemoglobin
EHR: Electronic health record
FEV1: Forced expiratory volume in the first second of the forced vital capacity
FVC: Forced vital capacity
GERD: Gastroesophageal reflux disease
H_2_B: Histamine‐2 receptor blocker
HTN: Hypertension
IPF: Idiopathic pulmonary fibrosis
LMAI: Leg movement arousal index
LMI: Leg movement index
LPR: Laryngopharyngeal reflux
Min SpO2: Minimum oxygen saturation recorded during sleep
N‐GSSIQ: Nocturnal Gastroesophageal Reflux Disease Symptom Severity and Impact Questionnaire
NIH: National Institutes of Health
NREM: Nonrapid eye movement sleep stage
OSA: Obstructive sleep apnea
PAP: Positive airway pressure
PFT: Pulmonary function test
PLM: Periodic leg movement
PLMAI: Periodic leg movement arousal index
PLMI: Periodic leg movement index
PPI: Proton‐pump inhibitor
PROMIS: Patient‐Reported Outcomes Measurement Information System
PSG: Polysomnography
RAI: Respiratory arousal index
RDI: Respiratory disturbance index
REM: Rapid eye movement sleep
SAI: Spontaneous arousal index
SDB: Sleep‐disordered breathing
SF‐36 RAND: Short Form 36 Health Survey
TAI: Total arousal index
TLC: Total lung capacity
TST: Total sleep time
UCSD SOBQ: University of California San Diego, Shortness of Breath Questionnaire
UW: University of Wisconsin
VA: Veterans affairs
WASO: Wake after sleep onset
WRS: Wilcoxon rank sum

## Ethics Statement

All procedures performed in this study were in accordance with the ethical standards of the University of Wisconsin (UW)–Madison, Wisconsin, Health Sciences Institutional Review Board (HS‐IRB #2019‐0891, approval date December 19, 2019), the William S. Middleton Memorial VA Medical Center, Madison, Wisconsin, Research and Development Committee (UW HS‐IRB #2021‐0959 and VA IRBNet #1638735; approval date March 17, 2022), and the 1964 Helsinki declaration and its later amendments.

## Consent

Informed consent was obtained from all participants in the study.

## Disclosure

The abstract was presented at American Thoracic Society International Conference—San Francisco, California, May 17^th^, 2022: https://www.atsjournals.org/doi/abs/10.1164/ajrccm-conference.2022.205.1_MeetingAbstracts.A5002. All co‐authors approved its final version. The content of this article is solely the responsibility of the authors and does not represent the views of the Department of Veterans Affairs or the United States Government.

## Conflicts of Interest

Mihaela Teodorescu reports funding support for this work from Boehringer Ingelheim Pharmaceuticals, Inc. The other authors declare no conflicts of interest.

## Author Contributions

M.T. is the guarantor of this manuscript. M.T. was responsible for conceptualizing the study; M.T., A.P., B.E., D.M., M.B., S.H., A.M., and N.S. were responsible for patient enrollment; A.P., B.E., D.M., I.M., L.M., and M.T. were responsible for data collection, processing, and management; M.T., B.E., and R.G. analyzed the data; B.E. and M.T. drafted the manuscript; and all co‐authors participated in data interpretation and provided critical review of the manuscript.

## Funding

This study​ was funded by an Investigator Initiated Award from Boehringer Ingelheim Pharmaceuticals, Inc. (US IIS_2018_00001353), with additional resources from the University of Wisconsin–Madison, Wisconsin, United States (administrative and facilities), and William S. Middleton Memorial Veterans’ Hospital, Madison, Wisconsin, United States (facilities). The authors meet the criteria for authorship as recommended by the International Committee of Medical Journal Editors (ICMJE). This was an independent, investigator‐initiated study supported by Boehringer Ingelheim Pharmaceuticals, Inc. (BIPI). BIPI had no role in the design, analysis, or interpretation of the results in this study; BIPI was given the opportunity to review the manuscript for medical and scientific accuracy as it relates to BIPI substances, as well as intellectual property considerations. The content of this article is solely the responsibility of the authors and does not represent the views of the Department of Veterans Affairs or the United States Government.

## Supporting Information

Figure S1: Relationships of N‐GSSIQ scores with dichotomous daytime symptoms (sleepiness, fatigue, lack of energy, and tiredness).

Figure S2: No relationships of clinically established GERD diagnosis with pulmonary and exercise physiology.

## Supporting information


**Supporting Information** Additional supporting information can be found online in the Supporting Information section.

## Data Availability

The data used and/or analyzed during the current study are available on reasonable request. Requests for access can be sent to Lynn Tarpey, Privacy/FOIA Officer, William S. Middleton Memorial Veterans Hospital, Madison, WI. Phone: 608‐256‐1901 x11699, Email: MadisonFOIA@va.gov.
