# Supplementary material for: Gastroesophageal Reflux, Sleep‐Disordered Breathing, and Outcomes in Patients With Idiopathic Pulmonary Fibrosis
Source: Can Respir J. 2025 Dec 28;2025:4228567. doi: 10.1155/carj/4228567 (PMC12745840; doi:10.1155/carj/4228567)
Supplement: Supplementary file 1 — Supporting Information Additional supporting information can be found online in the Supporting Information section. [file CARJ-2025-4228567-s001.docx]

**Title:** GASTROESOPHAGEAL REFLUX, SLEEP-DISORDERED BREATHING AND OUTCOMES IN PATIENTS WITH IDIOPATHIC PULMONARY FIBROSIS

**[Online Supplement]**

Braden Ellis, BS^1^; Daniel Morris, BA^1^; Andrea Peterson, MS^1^; Isabella Marquetti, BS^1^; Luke Manietta, BS^1^; Mikal Borg, PA^1^; Stephen Halliday, MD, MSCI^1,2^, Amy Malik, MD^1^; Nathan Sandbo, MD^1^; Ronald Gangnon, PhD^3,4^; Christopher J. Francois, MD^5^; Mihaela Teodorescu, MD, MS*^1,2^

^1^Department of Medicine, University of Wisconsin School of Medicine and Public Health –Madison, Wisconsin, United States; ^2^William S. Middleton Memorial Veterans Hospital, Madison, Wisconsin, Unites States; ^3^Department of Population Health Sciences and ^4^Department of Biostatistics and Medical Informatics, University of Wisconsin School of Medicine and Public Health – Madison, Wisconsin, United States; ^5^Department of Radiology, Mayo Clinic, Rochester, Minnesota, Unites States.

***Corresponding author:**

Mihaela Teodorescu, MD

William S. Middleton Memorial VA Medical Center

2500 Overlook Terrace │D2212 │Madison, WI 53705

Email: [mt3@medicine.wisc.edu](mailto:mt3@medicine.wisc.edu)

ORCID: <https://orcid.org/0000-0002-4490-6926>.

**Figure Legends:**

**Figure S1.** Relationships of N-GSSIQ scores with dichotomous daytime symptoms (sleepiness, fatigue, lack of energy and tiredness).


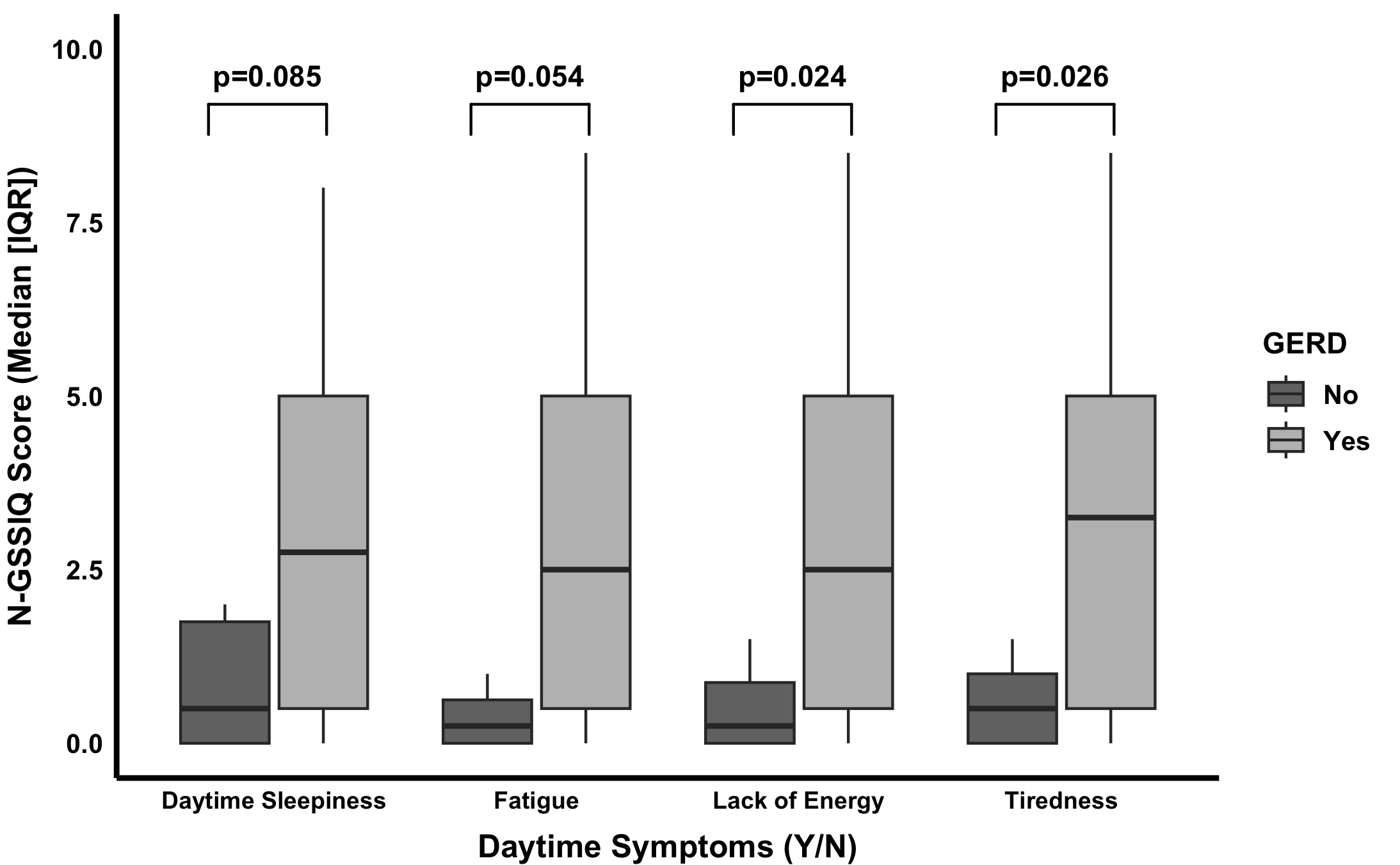


***Abbreviations:*** N-GSSIQ- Nocturnal Gastro-esophageal Reflux Disease Symptom Severity and Impact Questionnaire; IQR- Interquartile Range.

**Figure S2.** No relationships of clinically established GERD diagnosis with pulmonary and exercise physiology.


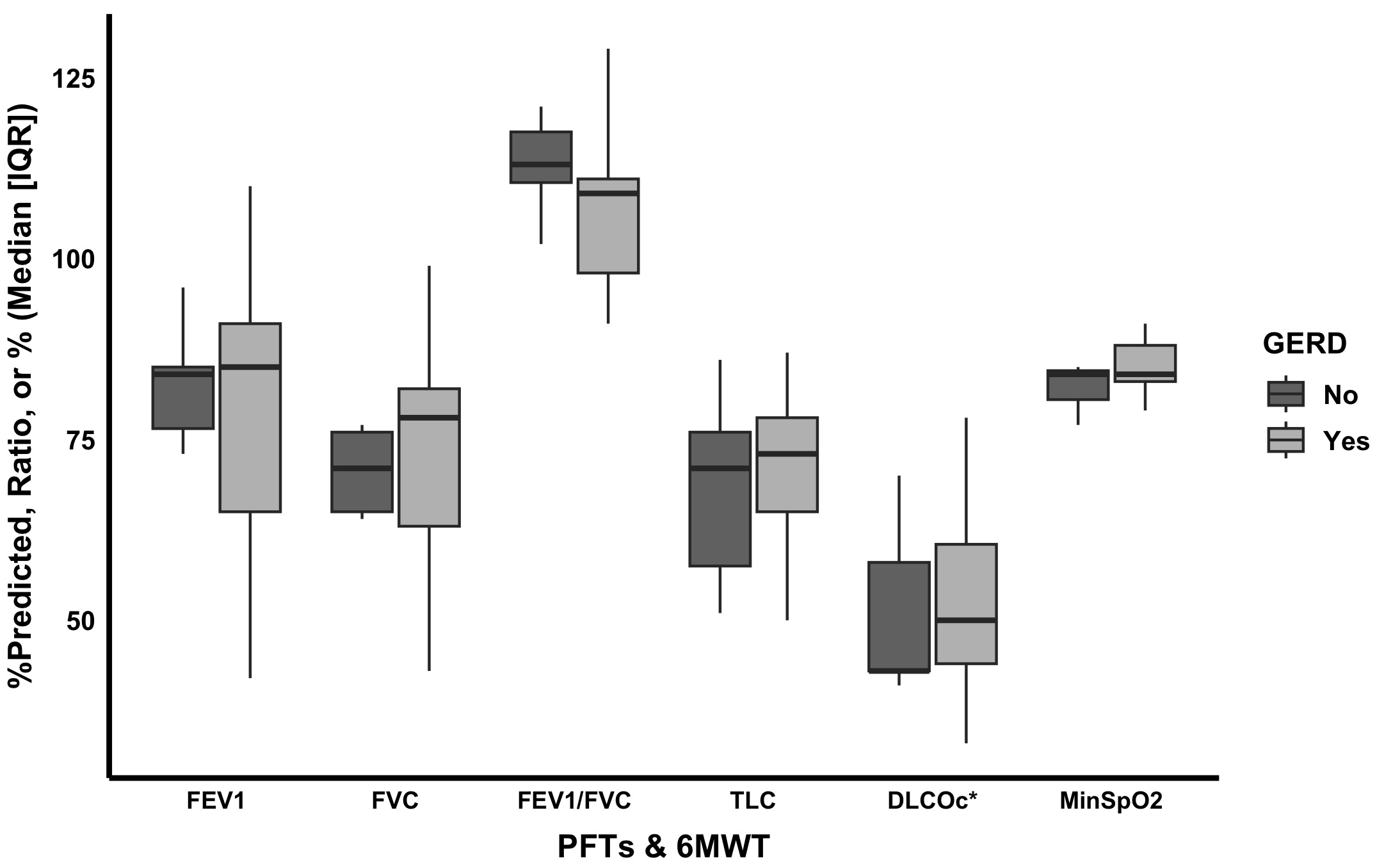


***Footnote:*** *Data available in N=20 subjects.

***Abbreviations:*** IQR- Interquartile Range; GERD- Gastro-esophageal Reflux Disease; FEV_1_-Forced Expiratory Volume in first second of forced vital capacity as percent of predicted; FVC- Forced Vital Capacity as percent of predicted; TLC: Total Lung Capacity as percent of predicted; DLCOc- Diffusing Capacity of the Lung for Carbon Monoxide corrected for hemoglobin, as percent of predicted; MinSpO_2_- Minimum O_2_ saturation during 6-Minute Walk Test; PFTs- Pulmonary Function Test; 6MWT- 6-Minute Walk Test.
